# Diagnostic intervals before and after implementation of cancer patient pathways – a GP survey and registry based comparison of three cohorts of cancer patients

**DOI:** 10.1186/s12885-015-1317-7

**Published:** 2015-04-23

**Authors:** Henry Jensen, Marie Louise Tørring, Frede Olesen, Jens Overgaard, Morten Fenger-Grøn, Peter Vedsted

**Affiliations:** 1 Research Centre for Cancer Diagnosis in Primary Care, Research Unit for General Practice, Department of Public Health, Aarhus University, Bartholins Allé 2, DK-8000 Aarhus C, Denmark; 2 Section for General Medical Practice, Department of Public Health, Aarhus University, Bartholins Allé 2, DK-8000 Aarhus C, Denmark; 3 Department of Clinical Medicine - Department of Experimental Clinical Oncology, Aarhus University Hospital, Noerrebrogade 44, DK-8000 Aarhus C, Denmark

**Keywords:** Diagnostic interval, Urgent referral, (early) diagnosis, Cancer, Primary care, Cohort, Denmark

## Abstract

**Background:**

From 2008, Danish general practitioners could refer patients suspected of having cancer to standardised cancer patient pathways (CPPs).

We aimed to compare the length of the diagnostic interval in 2010 with the length of the diagnostic interval before (2004/05) and during (2007/08) the implementation of CPPs in Denmark for all incident cancer patients who attended general practice prior to the cancer diagnosis.

**Methods:**

General practitioner questionnaires and register data on 12,558 patients were used to compare adjusted diagnostic interval across time by quantile regression.

**Results:**

The median diagnostic interval was 14 (95% CI: 11;16) days shorter during and 17 (95% CI: 15;19) days shorter after the implementation of CPPs than before. The diagnostic interval was 15 (95% CI: 12;17) days shorter for patients referred to a CPP in 2010 than during the implementation, whereas patients not referred to a CPP in 2010 had a 4 (95% CI: 1;7) days longer median diagnostic interval; the pattern was similar, but larger at the 75^th^ and 90^th^ percentiles.

**Conclusion:**

The diagnostic interval was significantly lower after CPP implementation. Yet, patients not referred to a CPP in 2010 tended to have a longer diagnostic interval compared to during the implementation. CPPs may thus only seem to expedite the diagnostic process for some cancer patients.

**Electronic supplementary material:**

The online version of this article (doi:10.1186/s12885-015-1317-7) contains supplementary material, which is available to authorized users.

## Background

Standardised cancer patient pathways (CPPs) have been introduced in some countries [1-8]. Even though CPP contents differ between countries, they all operate with a guaranteed timeframe for timely diagnosis. After years of increasing waiting times for cancer patients in Denmark, the Danish government and the Danish regions (i.e. hospital owners) declared in 2007 that cancer should be diagnosed and treated without delay [9]. Consequently, the Danish government and the Danish National Board of Health (today: the Danish Health and Medicines Authority) introduced CPPs in Denmark in 2008 [2]. CPPs were introduced in Denmark under the assumption that timely diagnosis and decisions on treatment options could be enhanced, psychosocial distress limited, and ultimately improve the prognosis for cancer patients. The Danish CPPs are standardised pathways for the time up to the final diagnosis and the start of treatment comprising well-defined sequences and time frames for diagnostic procedures and treatments for patients fulfilling CPP access criteria. The Danish CPPs were accompanied by renewal and expansion of equipment for imaging and radiotherapy. Patients can be referred to a Danish CPP when the clinician has *a reasonable suspicion of cancer as the final diagnosis* [2].

A key phase in the cancer journey is the diagnostic interval (DI), i.e. the time from the patient’s first presentation of symptoms in the health care system (usually in primary care) until diagnosis [10]. Despite a sparse body of evidence [11], mortality has been shown to increase with longer DI among patients with colorectal, lung, breast, melanoma or prostate cancers [12]. The DI is important as it measures the timeliness of the health-care system as a whole across sector boundaries.

Few studies have addressed the possible CPP impact on DI length. Most of these studies have primarily focussed on selected parts of the DI for specific cancer types, or studies have been performed with no baseline measures [1,4-7]. In addition, some studies are restricted to include only patients with predefined symptoms of cancer [13] or exclusively patients referred for a CPP [6] although some studies have reported that fairly few cancer patients initially present with well-known symptoms of cancer that allow direct access to a CPP [14,15]. Furthermore, many of the patients who are not referred to diagnostic workup through a CPP experience longer time intervals [7,16-18]. Thus, the possible effect of CPP implementation might vary with the actual use of CPPs, patient symptomatology and cancer types.

Therefore, we aimed to compare the length of the diagnostic interval in 2010 with the length of the diagnostic interval before (2004/05) and during (2007/08) the implementation of CPPs in Denmark for all incident cancer patients who attended general practice prior to the cancer diagnosis and for the five most common cancer types, regardless of the patients’ presenting symptoms.

## Methods

Data from GPs and registries from the Danish Cancer in Primary Care (CaP) cohort [19] was used in an ecological design to compare three cohorts of incident cancer patients before, during and after CPP implementation in order to investigate the impact of the CPP implementation in 2007–2009 [2] as a natural experiment. The ecological design was a consequence of the unknown joint distribution of CPP referral prior to the CPP implementation [20].

### Setting

The study took place in Denmark, where the annual cancer incidence rate is 326 per 100,000 [21]. The Danish publicly funded healthcare system ensures a uniform healthcare system with free access to diagnostics and treatment for all citizens. More than 98% of all citizens are registered with a specific general practice, which they may consult for medical advice. The Danish general practitioners (GPs) act as gatekeepers to the rest of the health care system. During the study period, 78.6% of all cancer patients in Denmark had been diagnosed through a primary care route [19].

### Patient population and data collection

Identification of patients, data collection and response analysis were based on the large Cancer in Primary Care study, which has been described in detail elsewhere [19]. In brief, we identified incident cancer patients aged 18 years or above and listed with a Danish GP and for whom diagnoses were coded according to the International Classification of Diseases, version 10 (ICD-10), i.e. C00.0-C99.9 (except for non-melanoma skin cancer (C44)), before from the former Danish County of Aarhus (640,000 inhabitants) (1 September 2004 – 31 August 2005), during from the Region of Southern Denmark and the Central Denmark Region (2.1 million inhabitants) (1 October 2007 – 30 September 2008) and after, from entire Denmark (1 May – 31 August 2010), the implementation of the CPPs at national level. Patients were identified through the Patient Administrative Systems (PAS) of the Danish hospitals and the Danish National Patient Registry. A patient was defined to have incident cancer when cancer was registered as the primary diagnosis during one of the inclusion periods, and no prior history of cancer was recorded. The history of cancer was checked in the Danish Cancer Registry [22]. We excluded 570 out of 22,736 patients (3%) because their diagnosis could not be verified by the Danish Cancer Registry.

For each sampled cancer patient, data from the registered GP was collected by a questionnaire, which was sent to the GP 2–5 weeks after identification of the patient. Participating GPs were asked to fill in the questionnaire on the basis of the information in their electronic medical records. Non-responders received a reminder after 3–5 weeks. Information from the questionnaire was combined with data from the Danish Cancer Registry to ensure that we obtained a validated date of diagnosis [22].

**Figure 1:**
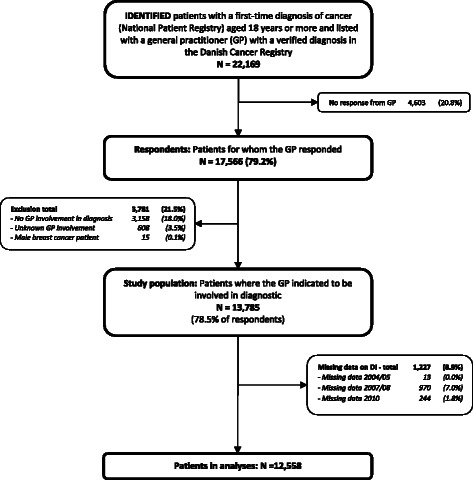
Flowchart for cancer patients. Boxes on the left indicate exclusion of patients who did not meet the inclusion criteria and boxes on the right indicate drop-outs due to non-response and missing data.

Figure 1 shows the patient flow in the present study. In 4,603 (20.8%) of the 22,169 verified cases, the invited GPs did not respond (GP response rate: 79.2%). Patients with responding GPs did not differ from patients with non-responding GPs in regard to 1-year survival, comorbidity or educational level. However, patients listed with responding GPs were more likely to be women, younger, to have a higher disposable income, to have more regionally or distantly spread tumours, and correspondingly more likely to have breast cancer and less likely to have prostate cancer than patients from non-responding GPs. These differences were small and clinically irrelevant, but were statistically significant due to the sample size (data published elsewhere) [19]. We excluded 3,766 (21.4%) patients from this study as the GP stated that (s)he was not involved in the diagnosis; i.e. patients diagnosed through screening, emergency access or as coincidental findings during diagnosis of other illnesses. We also excluded 15 male breast cancer patients (0.1%) (Figure 1).

### Outcome, exposure and possible confounders

In accordance with the Aarhus Statement, we defined the primary outcome, the DI, as the time from when the patient made the first symptom presentation to a GP until the time of diagnosis [10]. The DI was calculated by using the GP questionnaire to obtain the date of the patient’s first presentation of symptoms to the GP and the Danish Cancer Registry to define the date of diagnosis. The date of diagnosis recorded in this registry corresponds to the date of first contact (admission date) with the hospital department at which the cancer diagnosis was first registered as the primary cause of contact. If the patient was diagnosed by a private practicing specialist, the date of diagnosis corresponds to the date of the clinical diagnosis [23]. If the date of diagnosis was missing in Danish Cancer Registry, the admission date recorded in the Danish National Patient Registry was used.

Exposure was defined as implementation of CPP, and each of the three sub-cohorts was treated as an independent exposure group: 2004/05 = no CPPs implemented (before), 2007/08 = CPPs under implementation (during) and 2010 = fully implemented CPPs (after). Subsequently, we subdivided the ‘after’ group into two groups: patients who were initially referred to a CPP (after-CPP) and patients who were not (after-no CPP).

Possible confounders accounted for were gender, age, comorbidity, educational level and disposable income. Gender and age were derived from the patient’s Danish civil registration number [24]. We computed a Charlson Comorbidity Index score according to the method described by Quan et al. [25] using the date of the patient’s first consultation with the GP as the index date. We grouped the comorbidity scores into ‘none’ (no recorded disease), ‘moderate’ (score of 1 or 2), and ‘high’ (score of 3 or more). We grouped educational levels according to the International Standard Classification of Education (ISCED) [26] into ‘low’ (ISCED levels 1 and 2), ‘medium’ (ISCED levels 3 and 4) and ‘high’ (ISCED levels 5 and 6), and we categorized disposable household OECD income in the year prior to the diagnosis into tertiles (‘low’, ‘medium’ and ‘high’).

### Ethics

The study was approved by the Danish Data Protection Agency (file. no. 2009-41-3471). The Danish National Board of Health gave legal permission to obtain information from the GPs’ medical records. The study did not require approval from the Committee on Health Research Ethics of the Central Denmark Region as no biomedical intervention was performed.

### Analysis

We analysed changes in the DI for all cancers combined and for each of the five most common cancer types in Denmark: colorectal, lung, malignant melanoma, breast and prostate cancer [27].

All statistical analyses, except for analyses of missing data and sensitivity, were restricted to complete cases (i.e. GPs who completed the questionnaire and who were also involved in the diagnostic process).

Prefatory comparisons of the three sub-cohorts were performed using non-parametric methods: The Chi2 test was used for categorical data, while the Wilcoxon’s rank sum test was used for continuous data.

We used the qcount procedure written by Miranda [28] for the quantile regression analyses [29] on the smoothed quantiles to estimate the adjusted differences in the diagnostic interval at different percentiles; analysis on the smoothed quantiles are recommended for analyses of discrete (count) data [30]. Two adjusted models were considered: a model with no regard of referral route (overall trend) and a model with patients after the CPP implementation in 2010 divided into referral routes (trend by referral route). We adjusted for gender, age, cancer site, comorbidity, educational level and disposable income in both models. Age was centred at 45 years of age and was entered into the models as a continuous variable, while the other known confounders were entered as categorical variables.

To investigate the implication of missing data of DI, we performed best/worst case scenario sensitivity analyses by assigning the value 0 (best case) and the maximum values for the sub-cohorts (worst case) of the diagnostic intervals.

A statistical level of p ≤ 0.05 was considered significant in all analyses. Analyses were done using Stata® statistical software, version 13 (StataCorp LP, College Station, TX, USA).

## Results

### Demographic characteristics of excluded and included patients

In total 13,785 patients fulfilled the inclusion criteria. We excluded 1,227 patients (8.9%) due to missing information of the DI. These patients were more likely to be women, younger than 45 years of age or older than 75 years of age, to be diagnosed with breast or prostate cancers, to have high income or to have higher survival rates than the included patients. The characteristics of the analysed 12,558 are presented in Table 1.

**Table 1:** **Patient characteristics displayed by cohort time and total (N = 12,558)**

	Before	During	After			Total
			After-total	After-CPP	After-no CPP	
	n (%)	n (%)	n (%)	n (%)	n (%)	n (%)
**All**	2,041 (100)	6,660 (100)	3,857 (100)	1,432 (100)	2,425 (100)	12,558 (100)
**Gender**						
Male	981 (48.1)	3,305 (49.6)	2,051 (53.2)	729 (50.9)	1,322 (54.5)	6,337 (50.5)
Female	1,060 (51.9)	3,355 (50.4)	1,806 (46.8)	703 (49.1)	1,103 (45.5)	6,221 (49.5)
**Age groups (years):**						
18-44	176 (8.6)	490 (7.4)	300 (7.8)	106 (7.4)	194 (8.0)	966 (7.7)
45-54	232 (11.4)	771 (11.6)	464 (12.0)	171 (11.9)	293 (12.1)	1,467 (11.7)
55-64	479 (23.5)	1,640 (24.6)	862 (22.3)	336 (23.5)	526 (21.7)	2,981 (23.7)
65-74	562 (27.5)	1,924 (28.9)	1,162 (30.1)	432 (30.2)	730 (30.1)	3,648 (29.0)
75-	592 (29.0)	1,835 (27.6)	1,069 (27.7)	387 (27.0)	682 (28.1)	3,496 (27.8)
**Diagnoses**						
Colorectal	282 (13.8)	1,020 (15.3)	619 (16.0)	222 (15.5)	397 (16.4)	1,921 (15.3)
Lung	278 (13.6)	930 (14.0)	477 (12.4)	195 (13.6)	282 (11.6)	1,685 (13.4)
Melanoma, malignant	125 (6.1)	371 (5.6)	228 (5.9)	82 (5.7)	146 (6.0)	724 (5.8)
Breast	305 (14.9)	1,084 (16.3)	524 (13.6)	325 (22.7)	199 (8.2)	1,913 (15.2)
Prostate	213 (10.4)	858 (12.9)	560 (14.5)	220 (15.4)	340 (14.0)	1,631 (13.0)
Other	838 (41.1)	2,397 (36.0)	1,449 (37.6)	388 (27.1)	1,061 (43.8)	4,684 (37.3)
**Co-morbidity**						
None	1,519 (74.4)	5,008 (75.2)	2,912 (75.5)	1,101 (76.9)	1,801 (74.6)	9,439 (75.2)
Medium	432 (21.2)	1,429 (21.5)	786 (20.4)	278 (19.4)	508 (21.0)	2,647 (21.1)
High	90 (4.4)	223 (3.3)	159 (4.1)	53 (3.7)	106 (4.4)	472 (3.8)
**Educational level – ISCED**						
Low	754 (36.9)	2,820 (42.3)	1,430 (37.1)	547 (38.2)	883 (36.4)	5,004 (39.8)
Medium	709 (34.7)	2,345 (35.2)	1,506 (39.0)	537 (37.5)	969 (40.0)	4,560 (36.3)
High	386 (18.9)	1,161 (17.4)	770 (20.0)	293 (20.5)	477 (19.7)	2,317 (18.5)
Missing	192 (9.4)	334 (5.0)	151 (3.9)	55 (3.8)	96 (4.0)	677 (5.4)
**Disposable Income – OECD (EURO)**						
Low	639 (31.3)	2,113 (31.7)	1269 (32.9)	474 (33.1)	795 (32.8)	4,021 (32.0)
Medium	648 (31.7)	2,169 (32.6)	1313 (34.0)	466 (32.5)	847 (34.9)	4,130 (32.9)
High	662 (32.4)	2,185 (32.8)	1271 (33.0)	491 (34.3)	780 (32.2)	4,118 (32.8)
Missing	92 (4.5)	193 (2.9)	4 (0.1)	1 (0.1)	3 (0.1)	289 (2.3)

Column five and six display the ‘after cohort’ divided by referral route (referred to a Cancer Patient Pathway (after-CPP) or not (after-no CPP)).

### Diagnostic interval – overall tendency

**Figure 2:**
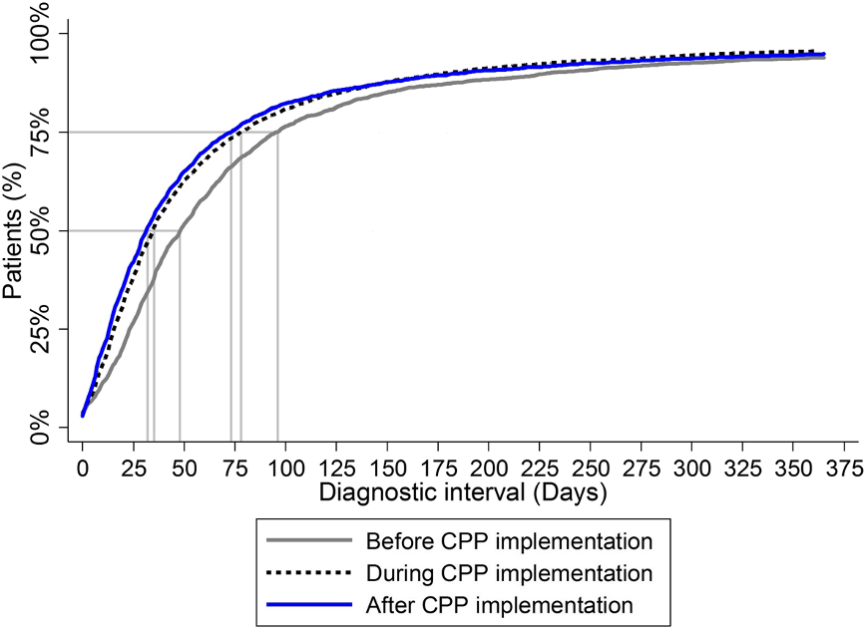
Cumulative frequencies of diagnostic interval (DI) before, during and after CPP implementation in Denmark. DI ranked in order and depicted by a Lorenz diagram. DI of longer than 365 days omitted for illustration purposes.

The unadjusted diagnostic intervals before, during and after CPP implementation are summarised in Table 2 and Figure 2. The median DI was statistically significantly lower across time: 49 (interquartile interval (IQI): 24;96) days before, 35 (IQI: 16;78) days during and 32 (IQI: 14;73) days after CPP implementation (Table 2 & Figure 2). The overall result remained the same when we adjusted for differences between populations; the median DI was 14 (95% CI: 11;16) days shorter during the transition stage than before CPP implementation and 17 (95% CI: 15;19) days shorter after CPP implementation (Table 3). Compared to the period before CPPs, the DI was shorter both during and after CPP implementation for all cancer types, although not statistically significant at all percentiles (Additional file 1).

**Table 2:** **The unadjusted diagnostic interval (DI) shown before (2004/05), during (2007/08) and after (2010 – combined) the implementation of CPPs (N=12,558)**

	Before	During	After		
			After-total	After-CPP	After-no CPP
	Median (IQI)	Median (IQI)	Median (IQI)	Median (IQI)	Median (IQI)
**Total**	49 (24;96)	35 (16;78)	32 (14;73)	22 (9;44)	41 (17;91)
**Gender**					
Female	41 (21;83)	31 (14;67)	29 (12;63)	18 (7;35)	39 (17;80)
Male	56 (28;118)	40 (19;89)	35 (14;82)	26 (12;54)	43 (18;102)
**Age groups**					
18-44 years	42 (21;93)	32 (15;69)	30 (13;72)	16 (8;29)	49 (21;92)
45-54 years	46 (22;84)	31 (15;64)	27 (13;55)	21 (8;34)	34 (16;64)
55-64 years	46 (23;87)	31 (15;71)	30 (13;64)	20 (8;41)	39 (18;80)
65-74 years	49 (25;98)	38 (18;82)	35 (15;78)	26 (12;53)	42 (17;95)
75 years and above	55 (26;107)	38 (16;91)	34 (13;84)	22 (8;53)	45 (17;109)
**Diagnoses**					
Colorectal	55 (27;95)	37 (17;84)	31 (14;69)	22 (8;46)	37 (18;80)
Lung	49 (27;85)	28 (12;64)	28 (11;67)	21 (9;47)	33 (12;80)
Melanoma, malignant	39 (23;67)	35 (17;69)	28 (12;55)	15 (6;29)	37 (19;74)
Breast	36 (19;56)	21 (13;36)	18 (8;34)	13 (5;23)	29 (15;51)
Prostate	85 (44;160)	57 (30;128)	46 (21;108)	34 (19;75)	53 (23;168)
Other	51 (22;113)	42 (18;95)	40 (16;88)	29 (11;56)	48 (18;100)
**Co-morbidity**					
None	49 (23;93)	34 (16;77)	31 (14;71)	21 (9;42)	41 (18;91)
Medium	49 (27;106)	38 (16;83)	34 (14;78)	26 (8;53)	41 (16;92)
High	43 (14;88)	35 (14;78)	31 (13;78)	19 (6;47)	46 (17;93)
**Disposable income – OECD**					
Low	50 (22;106)	36 (16;83)	32 (13;77)	22 (8;50)	42 (16;92)
Medium	49 (24;95)	35 (16;77)	32 (14;77)	22 (9;43)	41 (18;92)
High	50 (27;93)	34 (16;76)	31 (14;68)	22 (9;41)	41 (19;85)
Missing	32 (12;67)	34 (15;68)	50 (24;66)	3 (3;3)	55 (45;77)
**Educational level – ISCED**					
Low	49 (22;98)	35 (16;79)	32 (14;73)	23 (9;49)	42 (17;89)
Medium	47 (25;95)	34 (16;76)	32 (14;75)	21 (9;43)	40 (18;91)
High	51 (28;97)	36 (18;83)	31 (14;71)	22 (8;41)	42 (18;92)
Missing	49 (21;92)	29 (11;73)	32 (10;71)	24 (5;40)	40 (14;92)

Column five and six display the ‘after cohort’ divided by referral route (referred to a Cancer Patient Pathway (after-CPP) or not (after-no CPP)).

**Table 3:** **Estimated differences in diagnostic interval (DI) (calendar days) during and after the implementation of CPPs compared to before the implementation (Model 1), and also according to referral route after the implementation: to a CPP (after-CPP) or not (after-no CPP) (Model 2) (N=11,640)**

	Model 1^1^		Model 2^1^after-group split by referral to a CPP or not			
	During vs. before	After vs. before	After-CPP vs. before	After-no CPP vs. before	After-CPP vs. during	After-no CPP vs. during
	Estimate (95% CI)	Estimate (95% CI)	Estimate (95% CI)	Estimate (95% CI)	Estimate (95% CI)	Estimate (95% CI)
**Percentile**						
25^th^	**−7** (−8;-5)	**−10** (−11;-8)	**−12** (−13;-11)	**−6** (−8;-5)	**−8** (−9;-7)	0 (−2;1)
50^th^	**−14** (−16;-11)	**−17** (−19;-15)	**−23** (−25;-21)	**−9** (−12;-7)	**−15** (−17;-12)	**4** (1;7)
75^th^	**−22** (−27;-16)	**−27** (−34;-20)	**−46** (−51;-41)	**−11** (−21;-1)	**−32** (−37;-28)	**10** (1;19)
90^th^	**−53** (−76;-30)	**−44** (−65;-23)	**−110** (−153;-67)	−6 (−77;66)	**−80** (−126;-34)	48 (−49;145)

Estimates with 95% confidence intervals (95%CI) are displayed for the 25^th^, the 50^th^, the 75^th^ percentile and the 90^th^ percentiles. Bold estimates indicate statistical significance at p = 0.05 level or less.

Model 1 reference: before implementation group, cohort, female, 45 years of age, cancer sites, no co-morbidity, high disposable income and high educational level.

Model 2 = model 1, but with ‘after group’ split by referral route (CPP).

^1^Adjusted for gender, age, cancer site, co-morbidity, educational level and disposable income.

### Diagnostic interval by referral route compared to before CPP implementation

When patients diagnosed after CPP implementation were categorised according to the GPs use of CPP 62.8% were categorised as non-CPP referrals. The unadjusted median DI was lower for both the after-CPP group and the after-no CPP group compared to before the implementation of CPPs (p < 0.001) (Table 2). The DI was significantly longer for the after-no CPP group than for the after-CPP group (p < 0.001). This was observed for all the five major cancer types (Table 2). The 75^th^ percentile was 91 days in 2010 for the after-no CPP group compared to 44 days for the after-CPP group.

For the after-CPP group, the adjusted median was 23 (95% CI: 21;25) days shorter than before the implementation. For the after-no CPP group, the adjusted median was 9 (95% CI: 7;12) days shorter than before the implementation. At the 90^th^ percentile, the DI for the after-CPP group was 110 (95% CI: 67;153) days shorter than before, while similar (6 (95% CI: −66;77) days shorter) for the after-no CPP group than before (Table 3). This tendency was observed for all five major cancer types, although not statistically significant at all percentiles (Additional file 1).

### Diagnostic interval by referral route compared to during CPP implementation

For the after-CPP group, the adjusted median DI was 15 (95% CI: 12;17) days shorter than during the implementation. For the after-no CPP group, the adjusted median DI was 4 (95% CI: 1;7) days longer than during the implementation. Likewise, at the 90^th^ percentile, the DI for the after-CPP group was 80 (95% CI: 34;126) days shorter than during the implementation, while the DI for the after-no CPP group was insignificantly 48 (95% CI: −49;145) days longer than during the implementation (Table 3). This tendency was observed for all cancer types separately, although not statistically significant at all percentiles (Additional file 1).

### Sensitivity analysis

The sensitivity analyses did not alter the overall results as the median DI was still lower both during and after the implementation compared to before; this was found for both worst and best case scenario. Furthermore, both scenarios displayed that the median DI was longer for the after-no CPP group than for the after-CPP and also showed that the after-no CPP group tended to experience longer DIs after than during CPP implementation. Sensitivity analyses restricted to the same geographical region showed similar results.

## Discussion

We found that the median length of the DI in Denmark was shorter after the CPP implementation (in 2010) than before the CPP implementation (in 2004/05); the largest difference was found between the period before the CPPs (2004/05) and during the implementation phase in 2007/08. Furthermore, the largest difference in DI (compared to before the implementation) was found among patients in the after-CPP group, whereas patients in the after-no CPP group had only a minimally lower DI (compared to before the implementation) and still a long DI. In fact, the after-no CPP group in 2010 displayed a longer median DI than during the implementation. Finally, we found that the 90^th^ percentile DI for the after-no CPP group did not differ from before the CPP implementation. Hence, only patients in the CPP group in 2010 had a lower diagnostic interval after the CPP implementation than before CPP.

This finding must be compared to the fact that 63% of all Danish cancer patients in 2010 were not initially referred to a CPP [18]. Hence, the majority of cancer patients did not experience a lower DI across the investigated time period. This may have major impact on the prognosis for both patients with long DIs and at a population level, as it is reasonable to assume that expedited diagnosis of symptomatic cancer is likely to benefit the patients in terms of improved survival [12,31-35]. Hence, reductions in the diagnostic intervals (as we have shown) may influence the cancer stage distribution and hence survival at population level; these relations have been claimed to partly explain the improvement in survival among Danish cancer patients [36,37]. However, there is not yet enough evidence to substantiate this argument. Another equally important effect of reducing the diagnostic interval is that it should improve patient satisfaction and limit psychological distress among cancer patients, which was another important aim of the CPPS [2].

The literature on DI is sparse and direct comparisons between studies and countries are difficult [10]. We know of only two studies that have investigated the DI across time periods in connection with implementation of CPPs: a Danish study on head and neck cancer [5] and the large UK study on the implementation of the NICE guidelines [13]. Our results, which show a shorter DI after CPP implementation, are in line with the shorter DI found by these two studies. Yet, our study is the first to quantify adjusted changes in the DI across time at different percentiles. Our findings display that the decrease in the DI across time was largest among the patients who waited the longest, which may have major impact on the prognosis. Furthermore, we were able to quantify the changes in the DI across time by use of CPPs. We found that the patients referred to a CPP have a significantly shorter DI than patients not referred to a CPP at all percentiles. These findings are in line with the results of our previous studies in which we also accounted for the patients’ symptom presentation [18].

The design of the study does not permit us to infer causality between the implementation of CPPs and the lower DI seen across time. A number of changes in policy, clinical practice and investments may have contributed to these changes in DI across time. The implementation of CPPs was just one of many new initiatives in the second Danish cancer plan, which also promoted a huge expansion in radiotherapy facilities [38]. As the largest difference in DI was observed from before to during the implementation of CPPs, most of the differences found can probably not be attributed to the (full) implementation of the CPPs. The decision to implement CPPs was taken in August 2007, just after a declaration by the prime minister and the Danish National Board of Health that cancer should be treated without any delay in Denmark [9]. Therefore, local leaders may have started to streamline the diagnostic trajectories already before the official implementation in 2008/09, which could have contributed to the lower DIs observed in 2007/2008. Our study further shows that the general tendency towards lower diagnostic interval after the full implementation of the CPPs was different between patient referred to a CPP and patients who were not.

### Clinical implications

The results found in the study − together with other findings of longer intervals among patients not referred to rapid diagnostics [16,17] − supports the argument that introduction of CPPs only benefit patients referred to a CPP [17]. This has been suggested to be due to that the ‘fast-track’ system may disadvantage the large group of patients in whom the first appearance of disease does not involve significant cardinal symptoms of cancer [39,40]. This is underlined by the different proportion of CPP referrals between cancer types with e.g. breast cancer most often referred to a CPP [18]. Our findings may thus be interpreted as a demonstration of the possible danger of considering standardised CPPs as stand-alone referral routes for cancer. Our results may also indicate a need for an additional approach to ensure fast diagnosis of cancer, for instance by providing quick and easy access from primary care to all initial investigations ordered by a GP to establish the possibility of cancer [3].

### Strengths and weaknesses of the study

The main strength of this study is the study population, which was well-defined and complete with minimal selection bias [19,41]. We may have missed some patients, but this risk is expected to be negligible as we also included late-registered patients [41]. Another major strength that decreases the risk for selection bias is that we included all cancer patients, regardless of symptom presented at first contact and cancer site.

The Danish health care system is almost uniformly organized across different geographically and administratively independent regions. This organization allowed the merging of the three sub-cohorts into one, although they originated from partly overlapping geographical locations (regions) in Denmark and thus belonged to different subsets. In fact, the case-mix of the sub-cohorts resembles the case-mix in the DCR of a given year [41,42]. This indicates that the identified incident patients in the CaP Cohort are representative of incident cancer patients in Denmark at the time when the patients were identified.

The considerable size of the study ensures statistical precision, and the high response rate of 79% reduces the risk of selection bias. However, patients who were not included may have had longer DIs than the included patients. We believe this is not associated with the implementation of CPPs and, therefore, would not bias the observed DI differences between the sub-cohorts. Nevertheless, selection bias may still be present, but as our sensitivity analyses showed no impact on the results, this possible bias has been considered to be negligible (if present at all).

Information bias due to GP recall bias was reduced by using the GPs’ contemporaneously updated electronic medical records. Even so, the retrospective nature of the questionnaire holds the risk that the GPs may have misinterpreted the date of first presentation of symptoms for some of the cases. We believe that this possible risk is equal for all sub-cohorts and consequently will not bias the DI differences between the cohorts.

For obvious reasons, it is not possible to identify the patients in the before and during cohort, who would have been referred to CPPs had they been implemented. It is likely, that patients not eligible for CPPs would have had a tendency to longer DIs before and during the implementation. Hence, the fact that patients not referred to CPP have longer DI than the ‘during’ cohort as a whole, cannot be rigorously interpreted as a causal effect of CPPs disadvantaging this group. Furthermore, as the categorisation of patients according to CPP or not was based on the GPs choice of referral, comparison with all patients diagnosed at hospitals using CPPs must be cautioned.

The use of the date of first contact to a hospital ward as the date of diagnosis would tend to underestimate the length of the DI. This standard procedure is caused by the Danish Cancer Registry as the first contact date is recorded in this register as the date of diagnosis, even though most diagnoses are verified after this date (mostly at a multidisciplinary team meeting at the hospital). We consider this to be non-differential as we suspect that it is not associated with the CPP implementation, and hence deviations in date of diagnosis alone cannot explain the observed differences in DI between the cohorts. Yet, if this information bias may have been stronger for patients not referred to a CPP (after-no CPP group) as these patients have longer intervals and thereby may have raised the possibility that the date of diagnosis was moved relatively more than for the other groups of patients, this bias could have led to an underestimation of the observed DI difference between the after-no CPP group and the other groups. Our observed differences would thus be minimum estimates of the true differences.

## Conclusion

The diagnostic interval for the five most common cancers and for all cancers combined was lower in Denmark in 2010 than in 2004/05. The largest difference was seen from 2004/05 to 2007/08. Patients who were not referred to a CPP in 2010 still had long diagnostic intervals and tended to have a longer diagnostic interval than patients in 2007/08 when the CPP was not fully implemented. The patients with the 10% longest waiting time and who were not referred to a CPP in 2010 actually displayed a DI similar to the DI for the 10% waiting the longest in 2004/05.

These findings suggest that, despite the good intentions with implementing the CPPs, patients who were not referred to a CPP seem not to have gained faster diagnosis as these patients tend to have similar diagnostic intervals as before the implementation of CPPs. This demonstrates a need for more focus on providing faster diagnostic pathways for the large groups of patients who are not referred to a CPP in the initial phases of their disease.

## Additional file

Additional file 1:
**Estimated differences in diagnostic intervals (DIs) after and during CPP implementation compared to before, by cancer type.**

## Abbreviations

CPP: Standardised Cancer Patient Pathways
GP: General practitioner
CI: 95% Confidence Interval
CCI: Charlson Co-morbidty Index
ISCED: International Standard Classification of Education
