# Supplementary material for: Diagnostic intervals before and after implementation of cancer patient pathways – a GP survey and registry based comparison of three cohorts of cancer patients
Source: BMC Cancer. 2015 Apr 23;15:308. doi: 10.1186/s12885-015-1317-7 (PMC4412104; doi:10.1186/s12885-015-1317-7)
Supplement: Additional file 1: — Estimated differences in diagnostic intervals (DIs) after and during CPP implementation compared to before, by cancer type. [file 12885_2015_1317_MOESM1_ESM.pdf]

Addtional file: Estimated differences in diagnostic intervals (DIs) after and during CPP implementation compard to before, by cancer type.

Table A: Colorectal cancer: Estimated differences in diagnostic interval (DI) (calendar days) during and after the implementation of CPPs compared to before the implementation (Model 1). In addition, estimates are shown according to referral route after the implementation: to a CPP (after-CPP) or not (after-no CPP) (Model 2).
Estimates with 95% confidence intervals (95%CI) are displayed for the 25th, the 50th, the 75th percentile and the 90th percentiles. Bold estimates indicate statistical significance at p=0.05 level or less. (N=1,760)

	Model 1[1]				Model 2[1] – after-group split by referral to a CPP or not							
	during vs. before		after vs. before		after-CPP vs. before		after-no CPP vs. before		After-CPP vs. during		After-no CPP vs. during	
	Estimate	(95%CI)	Estimate	(95%CI)	Estimate	(95%CI)	Estimate	(95%CI)	Estimate	(95%CI)	Estimate	(95%CI)
25th percentile	**-8**	(-11;-5)	**-10**	(-12;-7)	**-14**	(-17;-11)	**-6**	(-10;-3)	**-10**	(-14;-6)	0	(-3;4)
50th percentile	-13	(-31;4)	**-18**	(-31;-5)	**-23**	(-37;-9)	-10	(-30;9)	**-15**	(-25;-6)	1	(-6;8)
75th percentile	**-12**	(-19;-6)	**-23**	(-42;-4)	**-45**	(-60;-31)	-10	(-23;3)	**-38**	(-53;-23)	1	(-15;17)
90th percentile	-4	(-122;115)	8	(-194;209)	**-79**	(-126;-32)	18	(-37;73)	**-73**	(-105;-41)	**27**	(1;54)

Model 1 reference: before implementation group, cohort, female, 45 years of age, cancer sites, no co-morbidity, high disposable income and high educational level.
Model 2 = model 1, but with after group split by referral route (CPP).
[1] adjusted for gender, age, cancer site, co-morbidity, educational level and disposable income

Table B: Lung cancer: Estimated differences in diagnostic interval (DI) (calendar days) during and after the implementation of CPPs compared to before the implementation (Model 1). In addition, estimates are shown according to referral route after the implementation: to a CPP (after-CPP) or not (after-no CPP) (Model 2).
Estimates with 95% confidence intervals (95%CI) are displayed for the 25th, the 50th, the 75th percentile and the 90th percentiles. Bold estimates indicate statistical significance at p=0.05 level or less. (N=1,539)

	Model 1[1]				Model 2[1] – after-group split by referral to a CPP or not							
	during vs. before		after vs. before		after-CPP vs. before		after-no CPP vs. before		After-CPP vs. during		After-no CPP vs. during	
	Estimate	(95%CI)	Estimate	(95%CI)	Estimate	(95%CI)	Estimate	(95%CI)	Estimate	(95%CI)	Estimate	(95%CI)
25th percentile	**-12**	(-14;-9)	**-11**	(-13;-9)	**-9**	(-13;-6)	**-10**	(-14;-6)	-2	(-5;1)	-2	(-10;7)
50th percentile	**-17**	(-23;-12)	**-17**	(-22;-12)	**-21**	(-25;-16)	-10	(-20;0)	**-9**	(-16;-2)	7	(-7;21)
75th percentile	**-26**	(-36;-15)	-18	(-40;3)	-34	(-74;6)	-6	(-29;16)	-15	(-62;32)	21	(-40;81)
90th percentile	-60	(-201;80)	-31	(-64;2)	**-56**	(-77;-35)	-24	(-58;11)	0	(-104;105)	44	(-18;106)

Model 1 reference: before implementation group, cohort, female, 45 years of age, cancer sites, no co-morbidity, high disposable income and high educational level.
Model 2 = model 1, but with after group split by referral route (CPP).
[1] adjusted for gender, age, cancer site, co-morbidity, educational level and disposable income

Table C: Malignant Melanoma: Estimated differences in diagnostic interval (DI) (calendar days) during and after the implementation of CPPs compared to before the implementation (Model 1). In addition, estimates are shown according to referral route after the implementation: to a CPP (after-CPP) or not (after-no CPP) (Model 2).
Estimates with 95% confidence intervals (95%CI) are displayed for the 25th, the 50th, the 75th percentile and the 90th percentiles. Bold estimates indicate statistical significance at p=0.05 level or less. (N=700)

	**Model 1[1]**				**Model 2[1] – after-group split by referral to a CPP or not**							
	**during vs. before**		**after vs. before**		**after-CPP vs. before**		**after-no CPP vs. before**		**After-CPP vs. during**		**After-no CPP vs. during**	
	**Estimate**	**(95%CI)**	**Estimate**	**(95%CI)**	**Estimate**	**(95%CI)**	**Estimate**	**(95%CI)**	**Estimate**	**(95%CI)**	**Estimate**	**(95%CI)**
25th percentile	-6	(-12;1)	**-10**	(-15;-5)	**-15**	(-17;-12)	-5	(-10;1)	**-12**	(-15;-10)	1	(-2;4)
50th percentile	-4	(-9;1)	**-11**	(-19;-4)	**-21**	(-27;-15)	0	(-11;11)	**-19**	(-24;-14)	4	(-6;13)
75th percentile	1	(-36;38)	-13	(-88;61)	-36	(-116;45)	7	(-102;115)	-37	(-94;20)	4	(-73;81)
90th percentile	**-100**	(-125;-75)	**-109**	(-122;-95)	**-116**	(-220;-12)	-56	(-134;21)	-90	(-188;7)	0	(-84;85)

Model 1 reference: before implementation group, cohort, female, 45 years of age, cancer sites, no co-morbidity, high disposable income and high educational level.
Model 2 = model 1, but with after group split by referral route (CPP).
[1] adjusted for gender, age, cancer site, co-morbidity, educational level and disposable income

Table D Breast cancer: Estimated differences in diagnostic interval (DI) (calendar days) during and after the implementation of CPPs compared to before the implementation (Model 1). In addition, estimates are shown according to referral route after the implementation: to a CPP (after-CPP) or not (after-no CPP) (Model 2).
Estimates with 95% confidence intervals (95%CI) are displayed for the 25th, the 50th, the 75th percentile and the 90th percentiles. Bold estimates indicate statistical significance at p=0.05 level or less. (N=1,811)

	**Model 1[1]**				**Model 2[1] – after-group split by referral to a CPP or not**							
	**during vs. before**		**after vs. before**		**after-CPP vs. before**		**after-no CPP vs. before**		**After-CPP vs. during**		**After-no CPP vs. during**	
	**Estimate**	**(95%CI)**	**Estimate**	**(95%CI)**	**Estimate**	**(95%CI)**	**Estimate**	**(95%CI)**	**Estimate**	**(95%CI)**	**Estimate**	**(95%CI)**
25th percentile	**-5**	(-6;-4)	**-9**	(-10;-7)	**-10**	(-12;-9)	-2	(-5;1)	**-7**	(-9;-5)	3	(-2;7)
50th percentile	**-11**	(-14;-8)	**-13**	(-15;-11)	**-16**	(-19;-13)	-4	(-8;0)	**-9**	(-13;-5)	**7**	(1;14)
75th percentile	**-18**	(-23;-14)	**-17**	(-20;-13)	**-25**	(-29;-21)	-6	(-11;0)	**-14**	(-16;-11)	**12**	(4;20)
90th percentile	**-24**	(-41;-8)	**-27**	(-45;-9)	**-42**	(-52;-32)	-6	(-27;15)	**-26**	(-34;-18)	19	(-8;46)

Model 1 reference: before implementation group, cohort, female, 45 years of age, cancer sites, no co-morbidity, high disposable income and high educational level.
Model 2 = model 1, but with after group split by referral route (CPP).
[1] adjusted for gender, age, cancer site, co-morbidity, educational level and disposable income

Table E Prostate cancer: Estimated differences in diagnostic interval (DI) (calendar days) during and after the implementation of CPPs compared to before the implementation (Model 1). In addition, estimates are shown according to referral route after the implementation: to a CPP (after-CPP) or not (after-no CPP) (Model 2).
Estimates with 95% confidence intervals (95%CI) are displayed for the 25th, the 50th, the 75th percentile and the 90th percentiles. Bold estimates indicate statistical significance at p=0.05 level or less. (N=1,553)

	Model 1[1]				Model 2[1] – after-group split by referral to a CPP or not							
	during vs. before		after vs. before		after-CPP vs. before		after-no CPP vs. before		After-CPP vs. during		After-no CPP vs. during	
	Estimate	(95%CI)	Estimate	(95%CI)	Estimate	(95%CI)	Estimate	(95%CI)	Estimate	(95%CI)	Estimate	(95%CI)
25th percentile	**-11**	(-18;-4)	**-20**	(-26;-13)	**-19**	(-25;-14)	**-16**	(-22;-9)	**-13**	(-16;-10)	**-7**	(-11;-3)
50th percentile	**-19**	(-27;-10)	**-29**	(-36;-22)	**-36**	(-41;-31)	**-22**	(-30;-13)	**-25**	(-30;-19)	-5	(-11;0)
75th percentile	**-34**	(-63;-6)	**-43**	(-56;-30)	**-73**	(-98;-49)	-2	(-85;81)	**-52**	(-81;-23)	32	(-54;119)
90th percentile	**-102**	(-152;-53)	-29	(-106;48)	**-244**	(-474;-15)	194	(-317;706)	**-195**	(-298;-91)	**305**	(65;545)

Model 1 reference: before implementation group, cohort, female, 45 years of age, cancer sites, no co-morbidity, high disposable income and high educational level.
Model 2 = model 1, but with after group split by referral route (CPP).
[1] adjusted for gender, age, cancer site, co-morbidity, educational level and disposable income

Table F Other cancer sites combined: Estimated differences in diagnostic interval (DI) (calendar days) during and after the implementation of CPPs compared to before the implementation (Model 1). In addition, estimates are shown according to referral route after the implementation: to a CPP (after-CPP) or not (after-no CPP) (Model 2).
Estimates with 95% confidence intervals (95%CI) are displayed for the 25th, the 50th, the 75th percentile and the 90th percentiles. Bold estimates indicate statistical significance at p=0.05 level or less. (N=4,277)

	Model 1[1]				Model 2[1] – after-group split by referral to a CPP or not							
	during vs. before		after vs. before		after-CPP vs. before		after-no CPP vs. before		After-CPP vs. during		After-no CPP vs. during	
	Estimate	(95%CI)	Estimate	(95%CI)	Estimate	(95%CI)	Estimate	(95%CI)	Estimate	(95%CI)	Estimate	(95%CI)
25th percentile	**-4**	(-7;-1)	**-6**	(-9;-3)	**-11**	(-15;-6)	**-4**	(-7;-1)	**-8**	(-13;-3)	0	(-3;4)
50th percentile	**-12**	(-19;-5)	**-13**	(-19;-7)	**-22**	(-27;-16)	-7	(-15;1)	**-15**	(-20;-9)	3	(-4;11)
75th percentile	-21	(-80;39)	-28	(-80;24)	**-54**	(-66;-43)	**-15**	(-26;-5)	**-42**	(-54;-29)	6	(-4;15)
90th percentile	-59	(-148;29)	**-49**	(-78;-20)	**-150**	(-187;-114)	-14	(-49;20)	**-121**	(-159;-83)	**44**	(14;74)

Model 1 reference: before implementation group, cohort, female, 45 years of age, cancer sites, no co-morbidity, high disposable income and high educational level.
Model 2 = model 1, but with after group split by referral route (CPP).
[1] adjusted for gender, age, cancer site, co-morbidity, educational level and disposable income
